# Development and validation of a nomogram to predict cancer-specific survival in elderly patients with papillary thyroid carcinoma: a population-based study

**DOI:** 10.1186/s12877-022-03430-8

**Published:** 2022-09-08

**Authors:** Jinkui Wang, Chenghao Zhanghuang, Liming Jin, Zhaoxia Zhang, Xiaojun Tan, Tao Mi, Jiayan Liu, Mujie Li, Xin Wu, Xiaomao Tian, Dawei He

**Affiliations:** 1 Department of Urology, Chongqing Key Laboratory of Children Urogenital Development and Tissue Engineering, Chongqing Key Laboratory of Pediatrics, Ministry of Education Key Laboratory of Child Development and Disorders, China International Science and Technology Cooperation Base of Child Development and Critical Disorders, National Clinical Research Center for Child Health and Disorders, Children’s Hospital of Chongqing Medical University, Chongqing, People’s Republic of China; 2 Department of Urology, Kunming Children’s Hospital, Yunnan Provincial Key Research Laboratory of Pediatric Major Diseases, Kunming, 650228 China

**Keywords:** Nomogram, Cancer-specific survival, Elderly patients, Papillary thyroid carcinoma, SEER

## Abstract

**Objective:**

Thyroid carcinoma (TC) is the most common endocrine tumor in the human body. Papillary thyroid carcinoma (PTC) accounts for more than 80% of thyroid cancers. Accurate prediction of elderly PTC can help reduce the mortality of patients. We aimed to construct a nomogram predicting cancer-specific survival (CSS) in elderly patients with PTC.

**Methods:**

Patient information was downloaded from the Surveillance, Epidemiology, and End Results (SEER) program. Univariate and multivariate Cox regression models were used to screen the independent risk factors for patients with PTC. The nomogram of elderly patients with PTC was constructed based on the multivariate Cox regression model. We used the concordance index (C-index), the area under the receiver operating characteristic curve (AUC) and the calibration curve to test the accuracy and discrimination of the prediction model. Decision curve analysis (DCA) was used to test the clinical value of the model.

**Results:**

A total of 14,138 elderly patients with PTC were included in this study. Patients from 2004 to 2015 were randomly divided into a training set (*N* = 7379) and a validation set (*N* = 3141), and data from 2016 to 2018 were divided into an external validation set (*N* = 3618). Proportional sub-distribution hazard model showed that age, sex, tumor size, histological grade, TNM stage, surgery and chemotherapy were independent risk factors for prognosis. In the training set, validation set and external validation set, the C-index was 0.87(95%CI: 0.852–0.888), 0.891(95%CI: 0.866–0.916) and 0.931(95%CI:0.894–0.968), respectively, indicating that the nomogram had good discrimination. Calibration curves and AUC suggest that the prediction model has good discrimination and accuracy.

**Conclusions:**

We constructed a new nomogram to predict CSS in elderly patients with PTC. Internal cross-validation and external validation indicate that the model has good discrimination and accuracy. The predictive model can help doctors and patients make clinical decisions.

## Background

Thyroid cancer (TC) is the most common endocrine tumor in the human body. According to the latest global cancer data report, TC ranks 11th among malignant tumors [1]. TC is also one of the fastest-growing malignancies, with the worldwide incidence of thyroid cancer increasing threefold in the past three decades and still growing at an annual rate of 3.6%. Ten years later, the incidence of thyroid cancer is expected to rise to fourth place [2]. This is mainly related to the increased sensitivity of color Doppler ultrasound and other examination items and the increase in the population census rate. However, several studies have found that the incidence of TC does increase after these factors are excluded [3–5]. Thyroid cancer mainly occurs in middle age, and older age is an independent risk factor for thyroid cancer prognosis. However, studies have shown that the 10-year cancer-specific survival rate of elderly TC patients can reach 60%-90% [6, 7]. Through literature review, we found that reports related to thyroid cancer were concentrated in children, adolescents and middle-aged people [8, 9], while few were in the elderly.

Papillary thyroid carcinoma (PTC) accounts for more than 80% of thyroid cancers, and most patients have a good prognosis. Studies have shown that microcarcinomas (< 10 mm) account for 33% of them [10]. These tumors often show a high degree of laziness and can obtain a good prognosis without surgical treatment. This microscopic carcinoma has a 30–60% autopsy rate but does not show any clinical symptoms or affect survival. Therefore, the American Thyroid Association (ATA) recommends against early intervention, including needle biopsy, radiographic follow-up, and surgical treatment for any minimal carcinoma [11]. However, the risk of recurrence and metastasis of thyroid cancer was linearly correlated with age, and the survival rate decreased with age. Vascular infiltration and early metastasis are more common in elderly patients [12]. In addition, studies have found that the frequency of lymph node metastasis and distant metastasis in elderly patients is also significantly higher [13]. More importantly, the survival rate of TC is as high as 92% in middle-aged patients, while it is only 48% in patients older than 70 years [12]. PTC combination is prone to early regional lymph node metastasis, and existing screening methods cannot accurately determine the aggressiveness of tumors. It is of great clinical value to accurately judge the aggressiveness of elderly PTC. In addition to avoiding excessive treatment and reducing hospitalization costs, it can reduce the mortality of elderly PTC patients and prolong the survival time of elderly patients.

Due to the low mortality rate of PTC patients, the importance of survival-related factors and other related factors is difficult to assess through single-center studies [14]. Park et al. [15] used machine learning to predict the survival prognosis of PTC patients. This model has good accuracy and practical value. Lin et al. [16] also used ultrasound-based risk stratification system to predict the risk of follicular thyroid tumors. This risk stratification system can accurately identify thyroid cancer. However, besides the traditional TNM staging system, no prediction model for cancer-specific survival (CSS) in elderly PTC patients has been developed and applied to our knowledge. The TNM staging system did not include crucial clinical information, such as sex, marital status, age, etc. [17], and could not validate the survival outcome [18].

We collected information from the National Cancer Institute's Surveillance, Epidemiology, and End Results Database (SEER) on elderly patients with PTC. We developed a nomogram to analyze and explore prognostic factors associated with CSS in the elderly PTC. We can predict the CSS rate of patients and provide a theoretical basis for clinical decision-making according to these characteristics.

## Patients and methods

### Data source and data extraction

We collected patient data from the SEER program to identify patients aged 65 years or older diagnosed with PTC between 2004 and 2018. Data for this study are available at http://seer.cancer.gov/. SEER data is the US National cancer database, containing approximately 30% of the US population and 18 cancer registries. The SEER database is a public database where patient data is publicly available and personal information is not identifiable. Therefore, our study does not require the approval of the ethics committee and the informed consent of patients. All methods included in this study comply with the published guidelines of the SEER database.

**Fig. 1:**
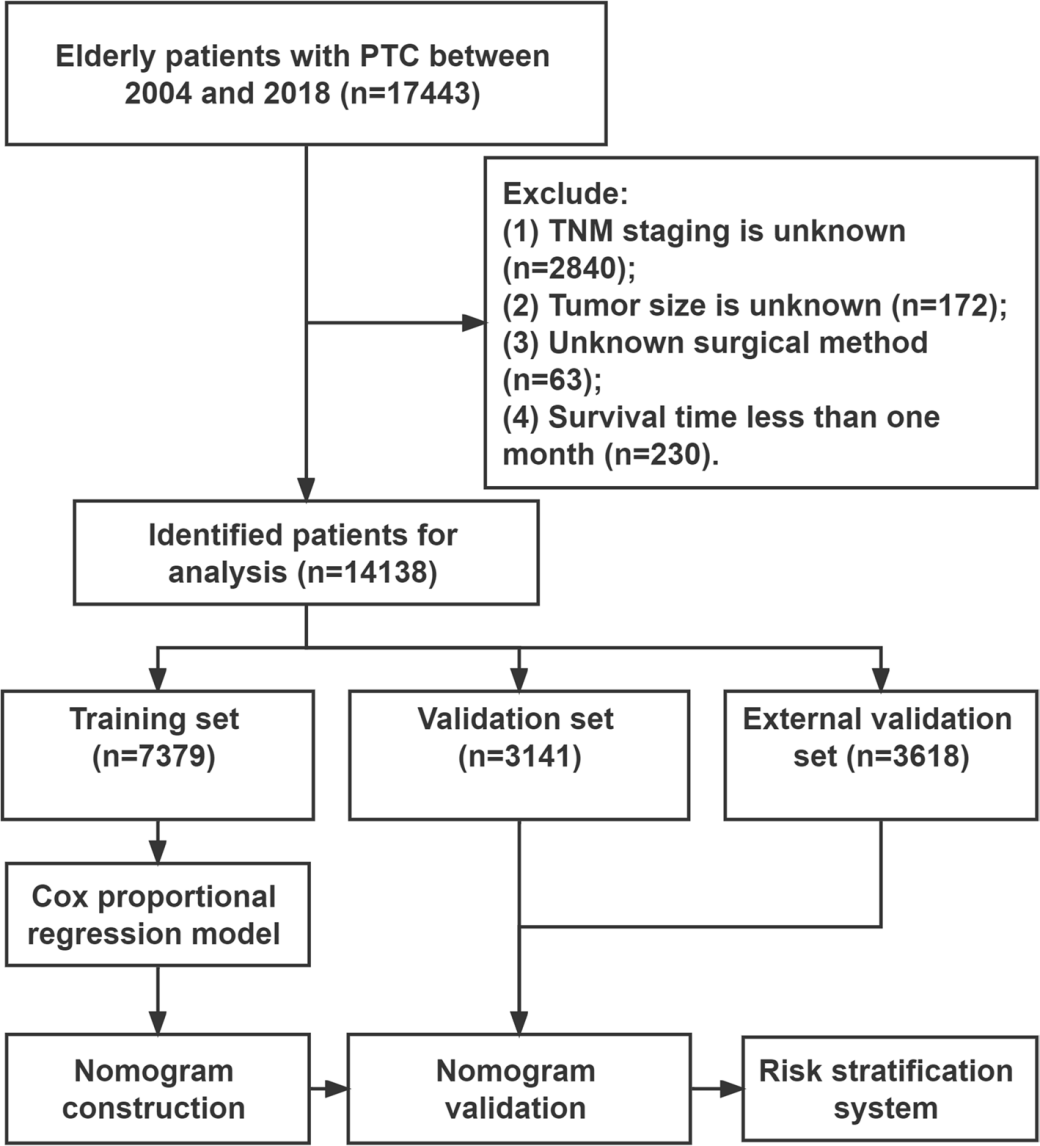
Flowchart for inclusion and exclusion of patients with PTC. Patients in the training set were used to screen independent risk factors and establish a nomogram. Validation set and external validation set were used to test the accuracy of the model. Finally, a risk stratification system is established to identify high-risk patients

We collected patients' demographic information (age, race, sex, year of diagnosis, marital status), clinicopathological information (histological tumor grade, tumor size, TNM stage), and treatment information (surgery, radiotherapy, chemotherapy), and follow-up results. Inclusion criteria:(1) age ≥ 65; (2) Pathological diagnosis of PTC; (3) The years of diagnosis were 2004–2018. Exclusion criteria:(1) tumor size is unknown; (2) TNM staging is unknown; (3) survival time less than one month; (4) The surgical method is unknown. The screening flow chart of all patients is shown in Fig. 1. In addition, in Fig. 1, we describe the construction and validation process of the nomogram.

Racial classifications include white, black, and other (American Indian /AK Native, Asian/Pacific Islander). Histological grading of tumors includes well-differentiated (I), moderately differentiated (II), poorly differentiated (III), and undifferentiated (IV). The marital status of the patients was either married or unmarried. The types of surgical procedures included nonoperative (code 0), thyroidectomy (code 10–30), subtotal or near-total thyroidectomy (code 40), and total thyroidectomy (code 50–80).

### Construction and validation of the competitive risk model

We first divided the patients into the 2004–2015 and 2016–2018 groups. The patients from 2004 to 2015 were used for the development and internal validation of the nomogram, and the patients from 2016 to 2018 were used for external validation in terms of time. Data from 2004 to 2015 were randomly divided into a training set (70%) and a validation set (30%). The cumulative risk model was used to estimate the cumulative incidence of cancer-specific death. Based on Fine and Gray's proportional sub-distribution hazard model, the influencing factors of cancer-specific death were analyzed [19, 20]. Proportional sub-distribution hazard model is a direct extension of Cox model in competitive risk situations. It is also based on semi-parametric proportional hazards, which can be derived from the cumulative risk model and used for multivariable analysis. We constructed a nomogram based on the competitive risk model for predicting CSS at 3-, 5-, and 10-year in elderly PTC patients. Calibration curves of 1000 bootstrap samples were used to validate the accuracy of the nomogram. In this study, discrimination refers to the ability to distinguish the survival or death of patients. Measured by the concordance index (C-index) [21], it is the area under the curve(AUC) of a receiver operating curve (ROC) [22, 23]. We used the C-index and AUC to test discrimination of the model.

### Clinical utility

A decision analysis curve (DCA) is a new algorithm to calculate the net benefit of the prediction model under different thresholds [24]. We used DCA to evaluate the clinical value of the nomogram prediction model. Also, we calculated the risk for each patient based on the nomogram. All patients were divided into high-risk and low-risk groups using the cut-off value of the receiver operating characteristic curve (ROC). Log-rank tests and Kaplan–Meier (K-M) curves were used to examine differences in survival among patients in each risk group. In addition, we analyzed the types of surgery performed by patients in different risk groups.

### Statistical analysis

Continuous variables (age, tumor size) were described by means and variances, and comparisons between groups were performed by chi-square or non-parametric U tests. Frequency (%) was used to describe categorical variables, and the Chi-square test was used to compare groups. Cox proportional regression model analyzed the prognostic factors of the patients. The survival differences were analyzed by log-rank test and K-M curve. All statistical analyses were performed by R software 4.1.0 and SPSS 26.0. A P value less than 0.05 was considered statistically significant.

## Results

### Clinical features

A total of 14,138 elderly patients with PTC were included in this study. Patients from 2004 to 2015 were randomly divided into a training set (*N* = 7379) and a validation set (*N* = 3141), and data from 2016 to 2018 were divided into an external validation set (*N* = 3618). Among the patients from 2004 to 2015, the mean age the patients was 72.4 ± 6.24 years, with 8804 (83.7%) white patients, 6163 (58.6%) married patients, and 3348 (31.8%) male patients. The mean tumor size of the patients was 18.0 ± 17.7 mm, with 6126 (58.2%) patients at the T1 stage, 8119 (77.2%) patients at the N0 stage, and 10,236 (97.3%) patients at the M0 stage. The histological tumor grades included 1580 (15.0%) patients in grade I, 416 (3.95%) in grade II, 143 (1.36%) in grade III and 97 (0.92%) in grade IV. 473 (4.50%) patients received non-operative, 1357 (12.9%) patients received lobectomy, 413 (3.93%) patients received subtotal or near-total thyroidectomy, 8277 (78.7%) patients received total thyroidectomy. 4525 (43.0%) patients received radiotherapy and 87 (0.83%) received chemotherapy. The clinicopathological information of patients showed no significant difference between the training set and the validation set (Table 1).

**Table 1:** Clinicopathological characteristics of elderly patients with PTC

	**ALL**	**Training set**	**Validation set**	
	***N* = 10,520**	***N* = 7379**	***N* = 3141**	**p**
Age	72.4 (6.24)	72.3 (6.21)	72.5 (6.30)	0.068
Race				0.647
White	8804 (83.7%)	6179 (83.7%)	2625 (83.6%)	
Black	505 (4.80%)	361 (4.89%)	144 (4.58%)	
Other	1211 (11.5%)	839 (11.4%)	372 (11.8%)	
Sex				0.824
Male	3348 (31.8%)	2343 (31.8%)	1005 (32.0%)	
Female	7172 (68.2%)	5036 (68.2%)	2136 (68.0%)	
Marital				0.312
No	4357 (41.4%)	3080 (41.7%)	1277 (40.7%)	
Married	6163 (58.6%)	4299 (58.3%)	1864 (59.3%)	
Year of diagnosis				0.044
2004–2009	4061 (38.6%)	2802 (38.0%)	1259 (40.1%)	
2010–2015	6459 (61.4%)	4577 (62.0%)	1882 (59.9%)	
Grade				0.907
I	1580 (15.0%)	1098 (14.9%)	482 (15.3%)	
II	416 (3.95%)	285 (3.86%)	131 (4.17%)	
III	143 (1.36%)	101 (1.37%)	42 (1.34%)	
IV	97 (0.92%)	68 (0.92%)	29 (0.92%)	
Unknown	8284 (78.7%)	5827 (79.0%)	2457 (78.2%)	
T				0.714
T1	6126 (58.2%)	4277 (58.0%)	1849 (58.9%)	
T2	1245 (11.8%)	873 (11.8%)	372 (11.8%)	
T3	2251 (21.4%)	1601 (21.7%)	650 (20.7%)	
T4	898 (8.54%)	628 (8.51%)	270 (8.60%)	
N				0.486
N0	8119 (77.2%)	5688 (77.1%)	2431 (77.4%)	
N1a	1350 (12.8%)	938 (12.7%)	412 (13.1%)	
N1b	1051 (9.99%)	753 (10.2%)	298 (9.49%)	
M				0.124
M0	10,236 (97.3%)	7192 (97.5%)	3044 (96.9%)	
M1	284 (2.70%)	187 (2.53%)	97 (3.09%)	
Tumor size	18.0 (17.7)	18.1 (18.0)	17.9 (16.9)	0.596
Surgery				0.255
No	473 (4.50%)	324 (4.39%)	149 (4.74%)	
Lobectomy	1357 (12.9%)	924 (12.5%)	433 (13.8%)	
Subtotal or near total thyroidectomy	413 (3.93%)	290 (3.93%)	123 (3.92%)	
Total thyroidectomy	8277 (78.7%)	5841 (79.2%)	2436 (77.6%)	
Chemotherapy				0.728
No/Unknown	10,433 (99.2%)	7316 (99.1%)	3117 (99.2%)	
Yes	87 (0.83%)	63 (0.85%)	24 (0.76%)	
Radiation				0.882
No/Unknown	5995 (57.0%)	4209 (57.0%)	1786 (56.9%)	
Yes	4525 (43.0%)	3170 (43.0%)	1355 (43.1%)	
Survival months	79.1 (41.4)	78.8 (41.2)	79.6 (42.0)	0.390

### Proportional sub-distribution hazard model analysis

Sub-distribution hazard model analysis showed that age, sex, race, tumor size, histological grade, TNM stage, surgery were the related factors for the survival and prognosis of patients. We also analysis the factors for the other cause of death, the results showed that age, sex, marital status, histological grade, NM stage, surgery, radiation and chemotherapy were independent risk factors for the prognosis of patients. Proportional sub-distribution hazard model analysis results are shown in Table 2.

**Table 2:** Proportional sub-distribution hazard models predict cancer-specific mortality (CSM) and other causes mortality (OCM) in elderly patients with PTC

	**CSM**			**OCM**		
	**HR**	**95%CI**	**P**	**HR**	**95%CI**	**P**
Age	1.044	1.029–1.06	< 0.001	1.081	1.072–1.089	< 0.001
Race						
white						
black	0.859	0.497–1.484	0.5	1.316	1.058–1.638	0.01
other	1.344	1.038–1.74	0.02	0.766	0.632–0.927	0.006
Sex						
Male						
Female	0.796	0.648–0.979	0.03	0.599	0.535–0.67	< 0.001
Marital						
No						
Married	0.849	0.699–1.032	0.1	0.838	1.193–0.936	0.002
Grade						
I						
II	1.383	0.837–2.286	0.21	0.834	0.612–1.137	0.3
III	3.369	2.055–5.525	< 0.001	0.585	0.352–0.971	0.038
IV	6.001	3.626–9.93	< 0.001	0.444	0.223–0.883	0.02
Unknown	1.216	0.882–1.676	0.23	0.896	0.773–1.039	0.2
T						
T1						
T2	2.036	1.429–2.902	< 0.001	1.053	0.887–1.251	0.6
T3	2.71	1.988–3.694	< 0.001	1.127	0.958–1.326	0.2
T4	5.263	3.719–7.449	< 0.001	1.016	0.81–1.273	0.9
N						
N0						
N1a	1.527	1.171–1.992	0.001	1.114	0.94–1.319	0.2
N1b	1.992	1.53–2.593	< 0.001	1.207	1.004–1.452	0.045
M						
M0						
M1	4.089	3.057–5.47	< 0.001	0.639	0.431–0.949	0.02
Tumor size	1.005	1.002–1.009	0.003	1.001	0.998–1.005	0.4
Surgery						
No						
Lobectomy	0.44	0.273–0.709	< 0.001	0.619	0.471–0.813	< 0.001
Subtotal or near total thyroidectomy	0.57	0.336–0.966	0.037	0.652	0.47–0.903	0.01
Total thyroidectomy	0.425	0.298–0.606	< 0.001	0.644	0.504–0.823	< 0.001
Chemotherapy						
No/Unknown						
Yes	1.6	0.962–2.663	0.07	0.638	0.305–1.334	0.2
Radiation						
No/Unknown						
Yes	0.977	0.783–1.22	0.84	0.794	0.706–0.893	< 0.001

### Nomogram construction for 3-year, 5-year, and 10-year CSS

**Fig. 2:**
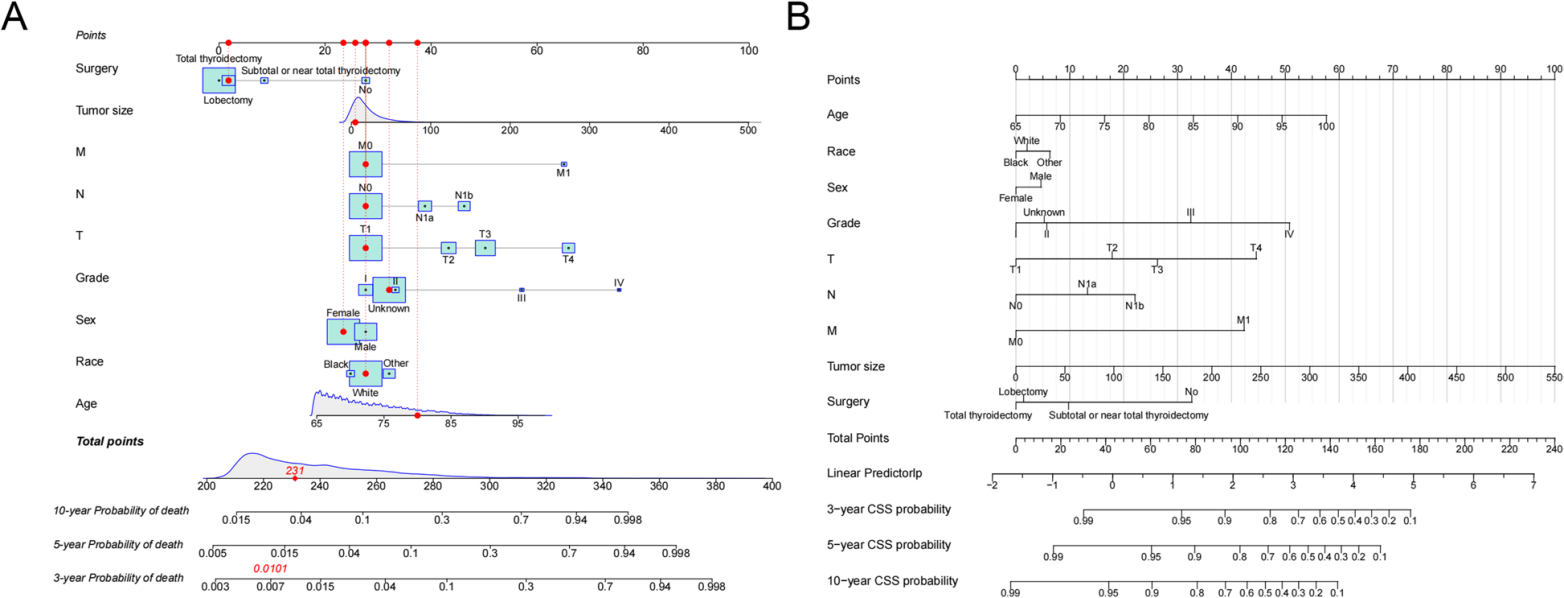
The nomogram for predicting 3-,5-,10-year CSS in elderly patients with PTC. **A **The first line is the scoring ruler, 2–10 rows are variables, and densities and boxes represent the distribution of the predictor variables in the sample. The total score of all variables corresponds to the probability of death at different time points. The red dot is the score and death probability of the sample patient; **B** Simplified version of the nomogram

We constructed a new nomogram based on proportional sub-distribution hazard model analysis to predict the CSS of elderly patients with PTC (Fig. 2). The figure shows that TNM stage and histological tumor grade were the most influential factors affecting patients' CSS. Treatment options, including surgery and chemotherapy, are also essential factors. Age and sex have less impact on patients.

### Validation of the nomogram

**Fig. 3:**
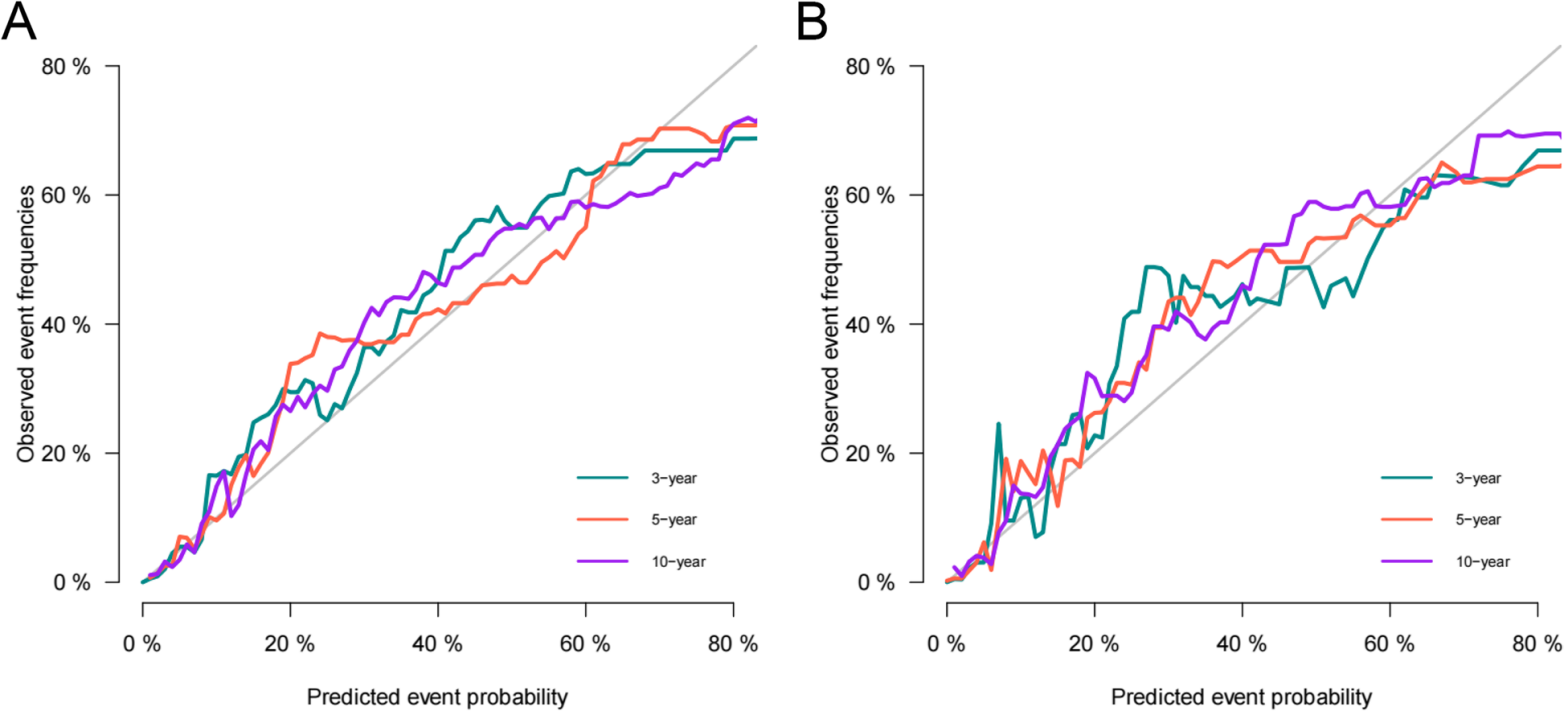
Calibration curve of the nomogram in the training set (**A**) and validation set (**B**). The horizontal axis is the predicted value in the nomogram, and the vertical axis is the observed value

**Fig. 4:**
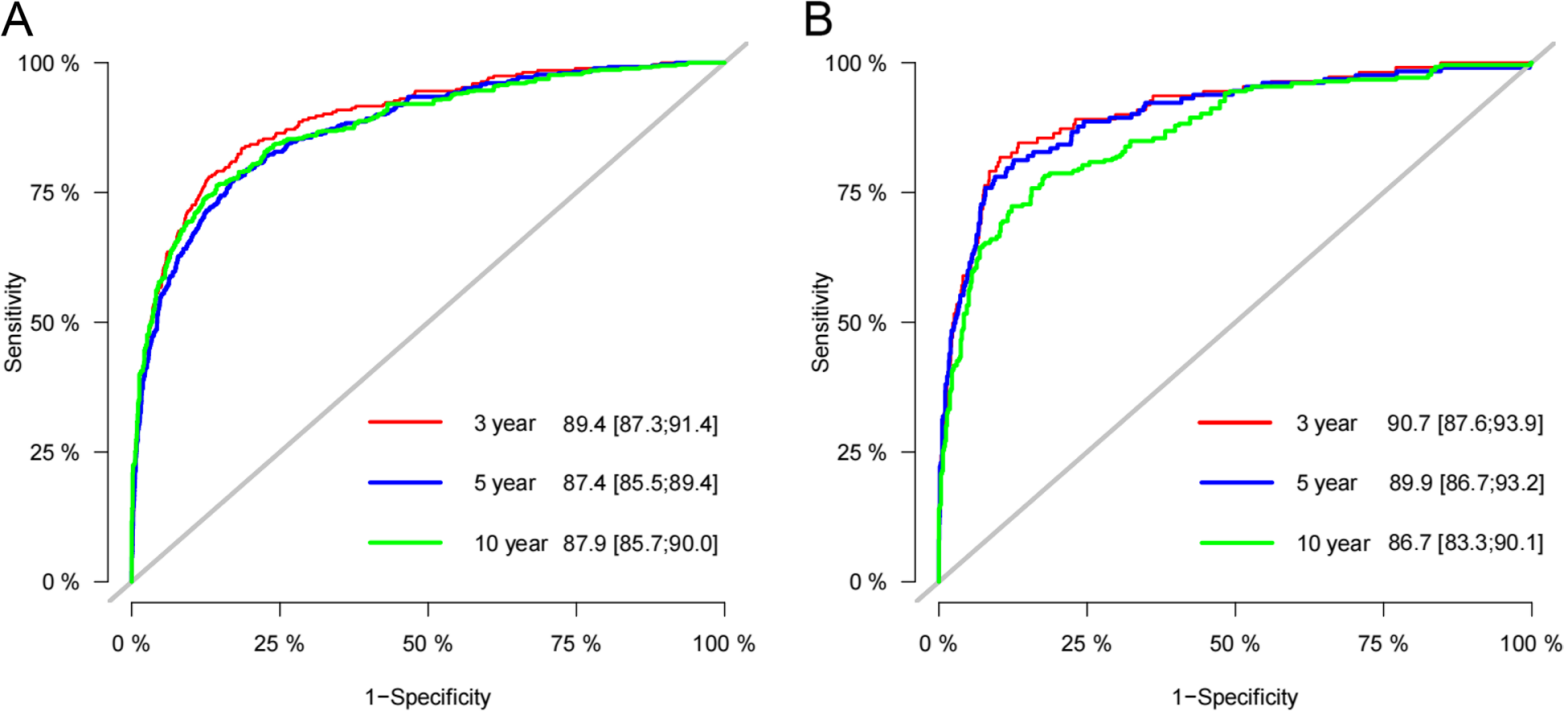
AUC for predicting 3-, 5-, and 10-year CSS in the training set (**A**) and validation set (**B**)

We use internal cross-validation and external validation to test the accuracy of the nomogram prediction model. We first used the C-index to test the model's discrimination. In the training set, validation set and external validation set, the C-index was 0.87(95%CI: 0.852–0.888), 0.891(95%CI: 0.866–0.916) and 0.931(95%CI:0.894–0.968), indicating that the nomogram had good discrimination. In the training and validation set, the predicted values on the calibration curve are highly consistent with the actual observed values (Fig. 3), indicating that the model has good accuracy. In the training set, the AUC of 3-, 5- and 10-year were 89.4, 87.4 and 87.9, respectively. In the validation set, the AUC at 3-, 5- and 10-year was 90.7, 89.9 and 86.7, respectively (Fig. 4). It shows that the prediction model has good discrimination.

### Clinical application of the nomogram

**Fig. 5:**
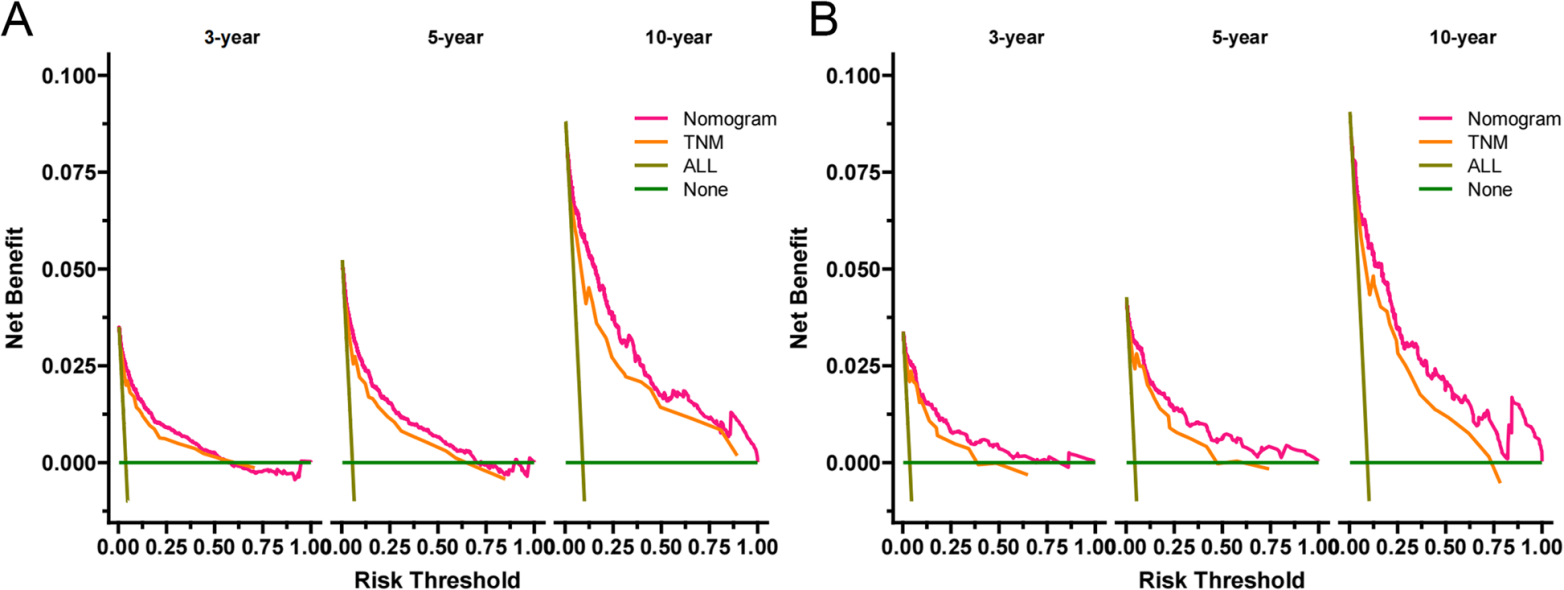
DCA of the nomogram in the training set (**A**) and the validation set (**B**). If the probability of death is high, it is necessary to treat. The Y-axis represents a net benefit, which quantifies the benefits of patients from treatment. The X-axis threshold probability indicates the probability of death of patients. The horizontal green line indicates that assuming all patients die, the net benefit of treatment is 0. The slanted dark green line indicates that assuming all patients survive, the net benefit decreases as the threshold increases. The yellow line was the traditional TNM tumor staging system, and the red line was the nomogram. When the threshold is between 0 and 100%, the clinical value of the nomogram is better than that of the traditional TNM staging, and it is significantly more beneficial than the assumed death or survival of all patients

**Fig. 6:**
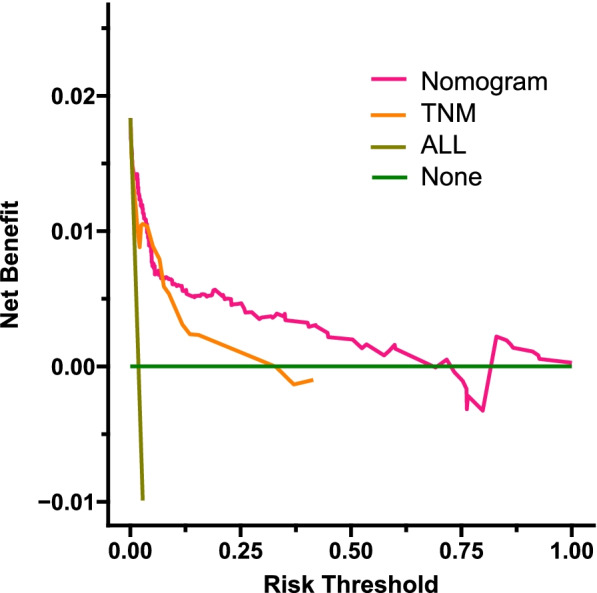
DCA of the nomogram in the external validation set

**Fig. 7:**
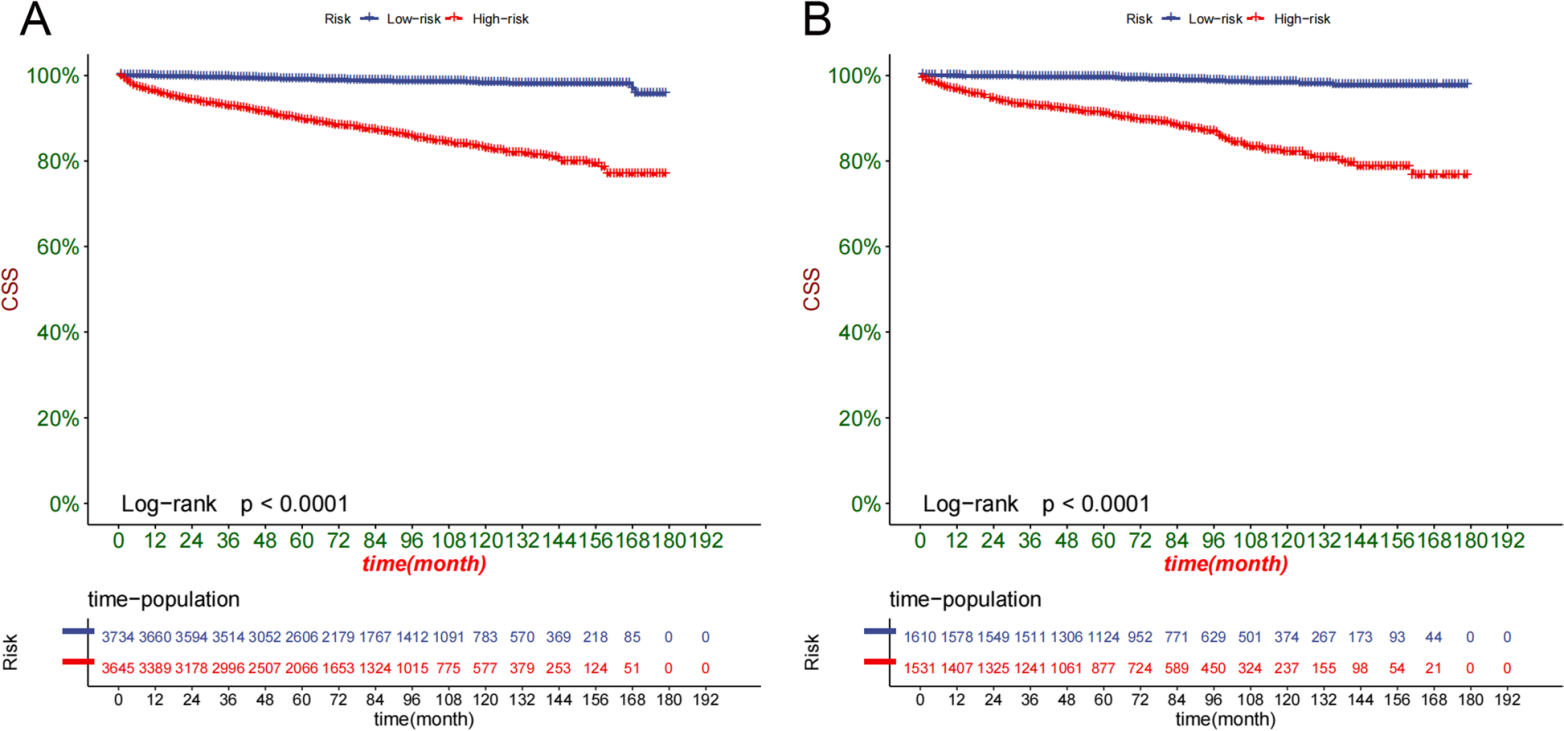
Kaplan–Meier curves of patients in the low-risk and high-risk groups in the training set (**A**) and validation set (**B**)

DCA results showed (Fig. 5) that the nomogram has excellent clinical utility value in both the training and validation set and the nomogram was superior to the traditional TNM staging. ROC cut-off values were used to divide patients into the high-risk group (total score ≥ 31.9) and the low-risk group (total score < 31.9). The external validation set DCA also showed that the model had good clinical value (Fig. 6). In both the training and validation sets, K-M curves indicated that the survival rate of patients in the high-risk group was significantly lower than that in the low-risk group (Fig. 7). 3-year, 5-year, and 10-year survival rates in the high-risk group were 92.7%, 90.0%, and 82.4%, respectively. In the low-risk group, 3-year, 5-year, and 10-year survival were 99.5%, 99.2%, and 98.2%, respectively.

**Fig. 8:**
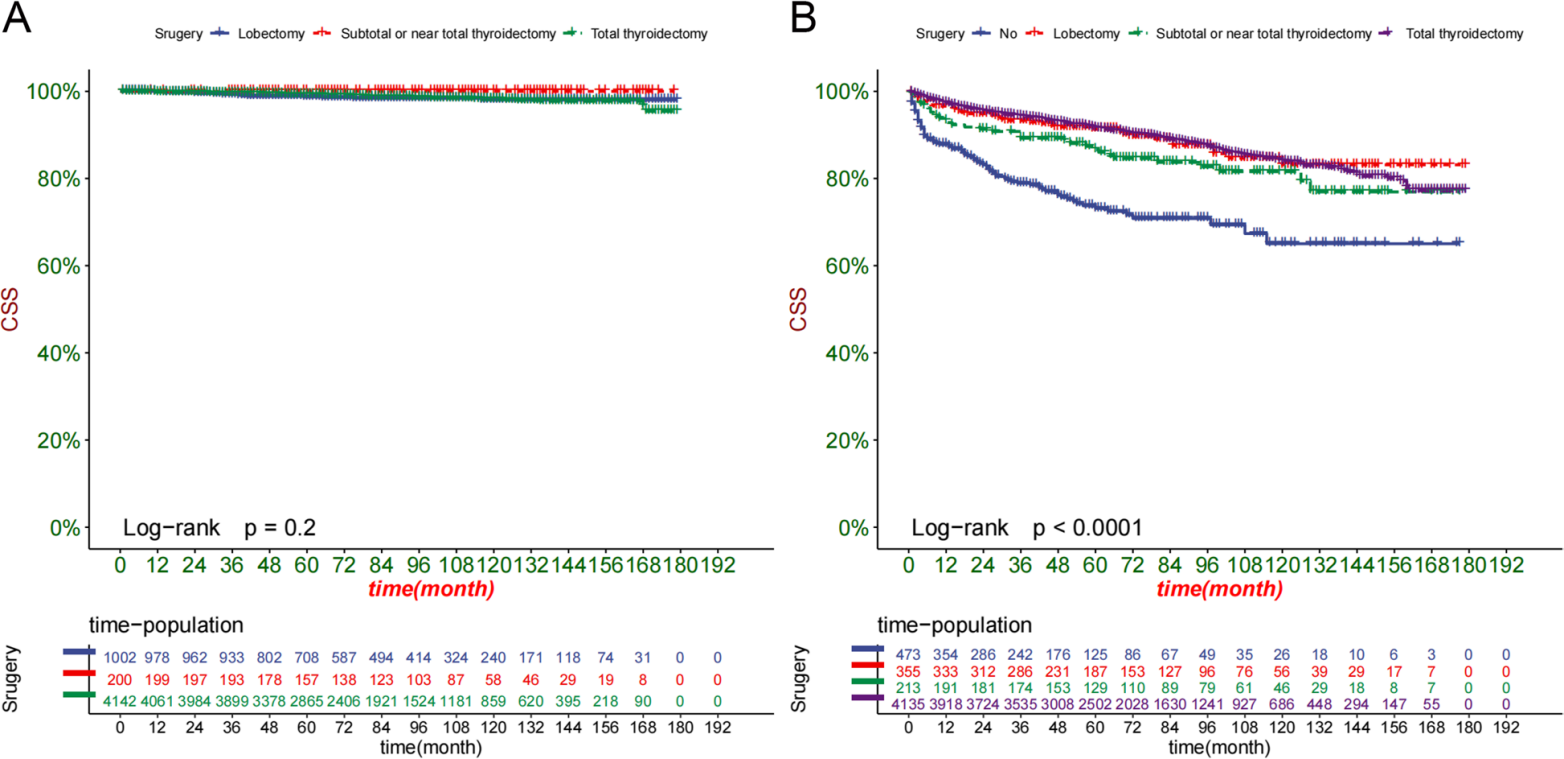
Kaplan–Meier curves of patients with different surgery in the low-risk group (**A**) and high-risk group (**B**)

In addition, we analyzed the type of surgery performed in the high-risk and low-risk groups (Fig. 8). We found that all patients in the low-risk group underwent surgery, and there were no significant differences between surgical procedures. In the high-risk group, most patients who received total thyroidectomy had the highest survival rates, while those with no surgery had the lowest.

## Discussion

Thyroid cancer is the most common malignant solid tumor of the head and neck, of which more than 80% are PTC. In addition, thyroid cancer is also one of the most heterogeneous tumors. Mutation analysis shows that mutations of different mutually exclusive genes can cause different types of thyroid cancer. Although most thyroid cancers have a good prognosis, there is a significant individual difference in prognosis [25]. Bilotti et al. [26] suggested that PTC may not be a clone from the same cell and that older and younger patients may be in two completely different forms rather than two different stages of the same disease. They also believe that older people are more resistant to the adverse effects of ionizing radiation. There are two completely different genetic mutations in elderly and young patients, resulting in two different PTC pathogenesis. Authoritative studies have shown that the malignant degree of thyroid nodules is significantly positively correlated with the elderly over 65 years old [27, 28]. In addition, PTC tends to represent larger tumor volume, more aggressive clinical features, and higher stage [29].

In many developed countries, we found that dividing old age by 65 is a widely accepted definition [30]. In this study, we defined the age standard of elderly patients as 65 years old and developed and validated the nomogram of elderly PTC patients. We found that the tumor size. TNM stage, tumor grade and age are independent risk factors for cancer-specific survival, consistent with most research results [31–34].

Age is associated with the prognosis of most malignancies, and thyroid cancer is only cancer in which age is included in staging to determine the prognosis of the thyroid. Studies have shown that the increase in age is linearly correlated with the recurrence, metastasis and mortality of PTC [35, 36]. Calo et al. found that the incidence of lymph node metastasis in young patients with PTC was nearly twice that of the elderly. However, the difference was not statistically significant, and the incidence of local infiltration was similar [37]. This study demonstrated that in older adults > 65 years of age, lymph node metastasis and distant metastasis were independent risk factors for predicting CSS. However, lymph node and hematogenous metastasis are very low in elderly PTC patients, with significantly distant metastasis.

Whether sex can be an independent risk factor for predicting TC prognosis remains controversial. Previous studies have shown that sex hormones can regulate thyroid growth. It is well known that estrogen has a protective effect on TC patients, although the specific mechanism remains unclear [38]. However, in our study, we did not find sex a risk factor for CSS, which may be because the analysis subjects were all over 65 years old, and women were postmenopausal with low estrogen levels. Banerjee et al. also suggested that only age and tumor characteristics determine overall survival (OS) in elderly patients with PTC. In addition, the male is only correlated with the prognosis of younger TC patients and does not affect survival after controlling the severity of the disease [14].

The nomogram relies on the close relationship between clinical information and prognostic outcomes as a big data prediction tool. Some studies have examined the findings of malignant thyroid tumors in the United States, and tumor size and prognosis of TC patients are in good agreement [39]. It is important to note that the treatment of minimal PTC with a tumor diameter of less than 1 cm is still controversial. In particular, recent evidence suggests that these microscopic PTC lesions rarely progress over time [40]. Since We cannot determine the aggressiveness of PTC, surgical resection is still preferred for incidental microcarcinoma, which increases the risk of postoperative complications caused by excessive treatment. A large number of studies have shown that in PTC patients, aggressive Thyroid Stimulating Hormone (TSH) inhibition and I-131 treatment do not reduce the risk of recurrence or improve OS [41–43]. More importantly, TSH suppression has been shown to cause patient harm, especially in elderly patients, including increased risk of atrial fibrillation, cardiovascular disease, and CSS, as well as osteoporosis in postmenopausal women [44], while I-131 damages normal thyroid tissue [45]. This study confirms that postoperative chemotherapy is a risk factor for CSS in elderly PTC patients rather than a protective factor. We suggest that aggressive TSH inhibition and I-131 therapy should not be recommended after PTC diagnosis, even in older adults older than 65 years.

Older patients have a shorter life span, higher rates of surgical complications, length of hospital stay, and non-cancer-specific mortality. It has been reported in the literature that the mortality rate caused by surgery reaches 7% for patients over 80 years old [46]. After evaluating age and colon cancer, gallbladder cancer, lung cancer and solid tumors of the head and neck, Korc-Grodzicki et al. concluded that surgery is the preferred treatment for solid malignant tumors, and age should not be a decisive factor in developing treatment strategies for patients [47]. Zhou et al. conducted propensity matching analysis on elderly PTC patients in the surgical and non-surgical groups. They showed that surgery would bring advantages to elderly PTC patients and recommended active surgical treatment for elderly patients under 85 years old [48]. Our study also found that surgical treatment was a protective factor for CSS in elderly PTC patients, and the prognosis was significantly better than that of the non-surgical group. However, it should be noted that there was no significant difference in outcome between total thyroidectomy, local thyroidectomy, and lobectomy.

However, there are some deficiencies in this study. First of all, the SEER database lacks smoking, alcohol consumption, BMI and other factors that may affect the CSS of elderly PTC patients. Secondly, as a retrospective study, selection bias may be unavoidable. Still, we included such critical factors as age, tumor size, sex, TNM stage and tumor grade, and the results would not be significantly biased. Thirdly, SEER database lacks comorbidity data, so the analysis results will have some deviations. Fourthly, the chemotherapy data in SEER database may be inaccurate or misleading, which may cause some deviation. Finally, although we performed external validation in terms of time, further prospective validation of our model in multi-center studies is required to confirm the model's accuracy.

## Conclusion

Our study found tumor size, surgery, TNM stage, tumor grade, and age were independent risk factors for CSS in elderly PTC patients. There was no correlation between sex and prognosis of elderly PTC. Finally, we established a new nomogram to predict CSS in elderly patients with papillary thyroid cancer. The model has been validated internally and externally with good accuracy and reliability, which provides a basis for clinical decision-making.

## Data Availability

The SEER data analyzed in this study is available at https://seer.Cancer.gov/.
